# Perplexing dynamics of *Wolbachia* proteins for cytoplasmic incompatibility

**DOI:** 10.1371/journal.pbio.3001644

**Published:** 2022-05-25

**Authors:** Toshiyuki Harumoto, Takema Fukatsu

**Affiliations:** 1 Hakubi Center for Advanced Research, Kyoto University, Kyoto, Japan; 2 Graduate School of Biostudies, Kyoto University, Kyoto, Japan; 3 Bioproduction Research Institute, National Institute of Advanced Industrial Science and Technology (AIST), Tsukuba, Japan; 4 Department of Biological Sciences, Graduate School of Science, The University of Tokyo, Tokyo, Japan; 5 Graduate School of Life and Environmental Sciences, University of Tsukuba, Tsukuba, Japan

## Abstract

The mechanism of symbiont-induced cytoplasmic incompatibility has been a long-lasting mystery. This Primer explores a new study on Wolbachia’s Cif proteins in PLOS Biology that provides supportive evidence for the “Host-Modification Model,” although the alternative “Toxin-Antidote Model” is still in the running.

## Teaser

The mechanism of symbiont-induced cytoplasmic incompatibility (CI) has been a long-standing mystery. A new study on *Wolbachia*’s Cif proteins in *PLOS Biology* provides supportive evidence for the “Host modification model,” although the alternative “Toxin–antidote model” is still in the running.

Cytoplasmic incompatibility (CI) is an elaborate strategy of some microbial symbionts, such as *Wolbachia*, *Cardinium*, and others, for driving their infections to spread into host populations. Typically, CI manifests as complete or partial offspring lethality specific to mating between infected males and uninfected females, which entails defective segregation of paternal chromosomes at the first mitotic division and subsequent developmental aberrations. Since the first recognition of symbiont-induced CI in *Culex* mosquitos in the 1970s, the molecular basis of CI has been elusive, with the abovementioned reproductive phenotypes being accounted for by “toxin–antidote,” “modification rescue,” or other conceptual hypotheses [1,2].

In 2017, this long-standing stasis was broken by identification of CI genes of *Wolbachia* strains *w*Mel of *Drosophila melanogaster* (*cifA* and *cifB*) and *w*Pip of *Culex* mosquitoes (*cidA* and *cidB*) [3,4]. Notably, transgenic *Drosophila* flies carrying the *Wolbachia*-derived genes were generated and mated with wild-type flies, by which means the host phenotypes analogous to CI were reproduced: The transgenic male flies suffered embryonic lethality of their offspring when mated with uninfected wild-type female flies [3,4] and restored normal embryonic development when mated with *Wolbachia*-infected wild-type female flies [3].

**Fig 1:**
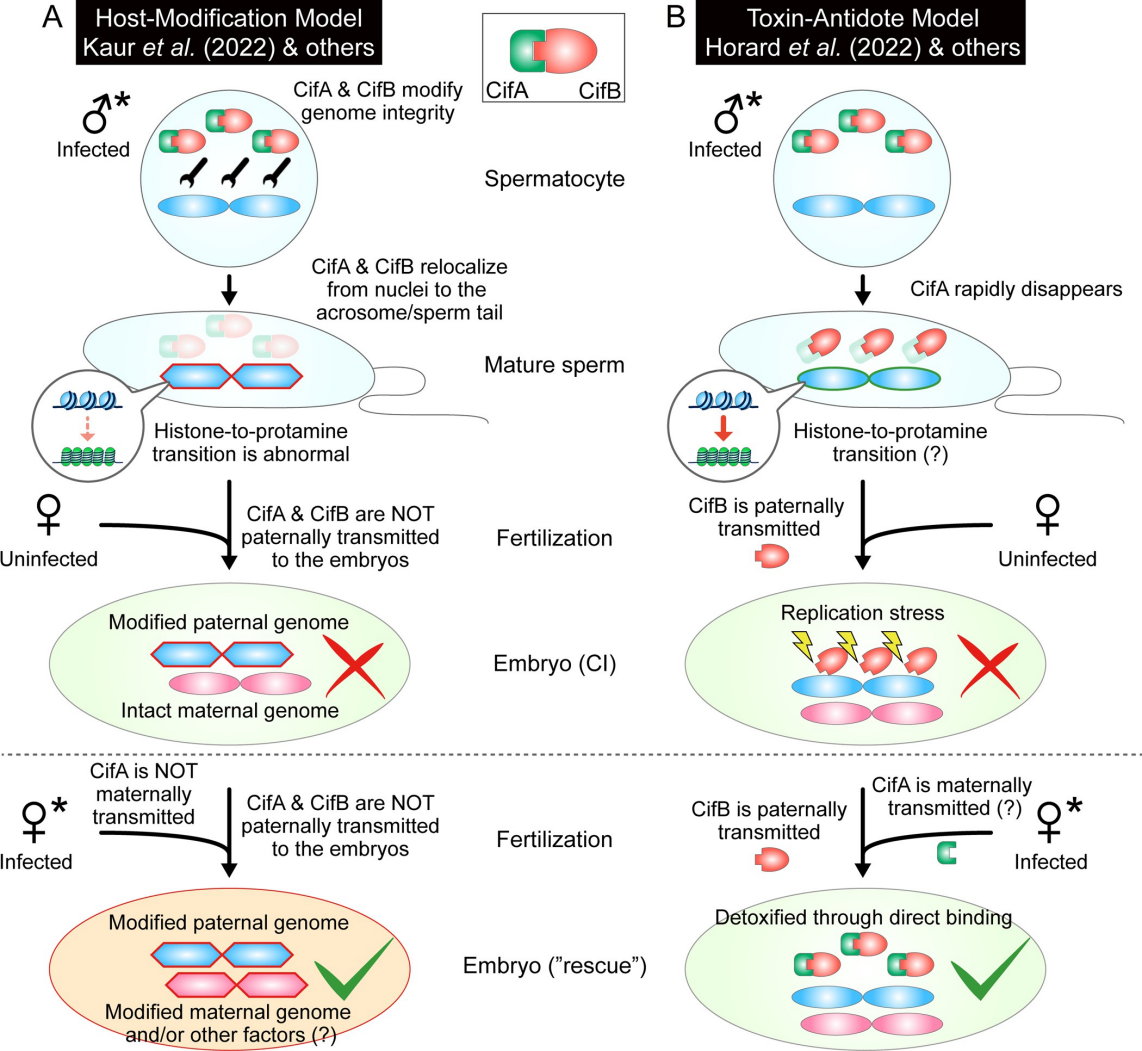
Two existing models on the mechanism of *Wolbachia*-induced CI. **(A)** The “Host modification model” supported by Kaur and colleagues [13] and others [2,3,5,6]. CifA and CifB temporarily localize to nuclei and modify paternal chromosomal integrity by altering the process of histone-to-protamine transition during spermatogenesis. In mature sperm, Cif proteins dissociate from nuclei and relocate to the acrosome and/or sperm tail. The paternal chromosomal modification is transmitted to fertilized eggs to induce CI, which is rescued by the female-derived modification caused by CifA during oogenesis. Note that Cif proteins themselves are not delivered to fertilized embryos and thus do not directly affect CI and rescue. **(B)** The “Toxin–antidote model” supported by Horard and colleagues [14] and others [4,7–11]. CifA and CifB colocalize during the early spermatogenesis. Despite their existence, the process of the histone-to-protamine transition exhibits no obvious defect. After the transition, CifA rapidly disappears while CifB is retained in maturing sperm nuclei and paternally transmitted to fertilized eggs to induce CI-associated phenotypes (replication stress). Rescue is underpinned by the direct binding of CifB to CifA, which is assumed to be maternally provided. CI, cytoplasmic incompatibility.

By the breakthrough-making studies, the previously conceptual CI factors were embodied as real genes and proteins, which boosted accumulation of information regarding the dynamics, interactions, and functioning of the CI proteins, called CifA (or CidA) and CifB (or CidB) [5–10] (hereafter, we use “Cif” for simplicity and readability). On the basis of these new data, the updated hypotheses—the “Host modification model” [2] (Fig 1A) and the “Toxin–antidote model” [11] (Fig 1B)—were proposed as the molecular mechanism of CI, although they remain controversial [11,12]. To address which of these contrasting models is more appropriate, one must visualize and track the in vivo distribution and dynamics of *Wolbachia*’s CI proteins in detail during the host’s gametogenesis and pre- and postfertilization processes.

In this issue of *PLOS Biology*, Kaur and colleagues [13] provide detailed immunocytochemical visualization of the CI proteins, CifA and CifB, of the *Wolbachia* strain *w*Mel during the early developmental course of wild-type and transgenic *D*. *melanogaster*. What they demonstrate are the following: In testis, (i) CifA and CifB localize to nuclear DNA throughout spermatogenesis; (ii) Cif proteins cause abnormal histone retention in elongating spermatids and protamine deficiency in mature sperm; and (iii) in mature sperm, CifA and CifB dissociate from nuclei and relocate to the acrosome and/or sperm tail. In ovary, (iv) CifA localizes to the germ cell nuclei and cytoplasm of early-stage egg chambers; (v) by contrast, CifA is not detected in late-stage oocytes; and (vi) Cif proteins remain undetectable in fertilized embryos. In addition, using protamine mutants of *D*. *melanogaster*, they demonstrate that (vii) protamine gene knockout results in increased protamine deficiency in mature sperm as well as enhanced intensity of CI. Furthermore, they identify a newly annotated bipartite nuclear localization signal (bNLS) sequence in CifA and demonstrate that (viii) bNLS of CifA plays a role in expression of CI and rescue. Taken together, these observations are generally concordant with the “Host modification model” of CI (Fig 1A). The experimental demonstration that the protamine deficiency in paternal chromosomes is linked to the intensity of CI is a seemingly important finding that provides insight into the molecular and cellular processes underpinning host modification. The discovery of the bNLS as a functional element of CifA needed for both CI and rescue is also an important finding, which is supportive of CifA functioning in both modification and rescue, the so-called “Two-By-One model” of CI [5,6].

However, unsolved mysteries still remain. Kaur and colleagues [13] report that Cif proteins are undetected in mature oocytes and fertilized embryos, suggesting that, although *cifA* is identified as the essential gene for rescue [5,6], CifA protein cannot directly participate in the rescuing action in fertilized embryos. What, then, is the direct agent of rescue? Also, Kaur and colleagues [13] highlight abnormal histone-to-protamine transition as a symptom relevant to paternal chromosomal modification, but the direct target of modification is still elusive. These issues should be addressed in future studies.

Notably, in contrast to the “Host modification model” (Fig 1A) supported by Kaur and colleagues [13] and others [2,3,5,6], there are several important studies that alternatively propose/support the “Toxin–antidote model” (Fig 1B) [4,7–11,14]. These studies, taken together, suggest that (i) CifB protein acts on host chromosomes and induces host lethality; (ii) CifA protein is capable of direct binding to/interaction with CifB protein; and (iii) the CifA binding to CifB suppresses the lethal action of CifB, which looks analogous to the toxin–antidote relationship. In particular, the demonstration of CifA–CifB binding using pull-down assay [4], yeast cells [4,9], insect culture cells [14], and crystallography [9,10] seems in favor of the “Toxin–antidote model” [11], although the CifA–CifB binding does not necessarily preclude and could be also integrated into the “Host modification model” [2,6].

Recently, Horard and colleagues [14] reported detailed immunocytochemical and transgenic visualization of CifA and CifB of the *Wolbachia* strain *w*Pip, which is comparable, although not perfectly, to the cytological work on CifA and CifB of the *Wolbachia* strain *w*Mel by Kaur and colleagues [13]. Here, we contrast the observations by Horard and colleagues [14] with those of Kaur and colleagues [13] in the context of the alternative CI models (Fig 1). In Horard and colleagues [14], while CifA and CifB localize to nuclear DNA during early spermatogenesis, CifA subsequently disappears and only CifB persists in maturing sperm nuclei and transmitted to the fertilized embryos, and upon fertilization, the CifB-associated paternal chromosomes fail to segregate at the onset of first mitosis (Fig 1B). In Kaur and colleagues [13], by contrast, not only CifA but also CifB is not transmitted to the fertilized embryos (Fig 1A). While Kaur and colleagues [13] report abnormal histone-to-protamine transition in mature sperm (Fig 1A), Horard and colleagues [14] observed no obvious defect in the progression of spermatogenesis, although more detailed inspection may be needed to verify the observation (Fig 1B). In these respects, our current knowledge is conflicting as well as perplexing. Why are some observations of Kaur and colleagues [13] distinct from those of Horard and colleagues [14]? Is this solely due to the difference between *Wolbachia* strains *w*Mel and *w*Pip? Or is it because of differences between the originally endogenous Cif proteins and the foreign transgenic Cif proteins under the *D*. *melanogaster* background? Or could other potential factors be in operation?

In conclusion, although the main players involved in the *Wolbachia*-induced CI have been identified as CifA and CifB, it has not yet been established how these proteins interact with each other, with paternal chromosomes, with developing oocytes, and with fertilized embryos, thereby accounting for the dramatic host phenotypes of CI. The current observations, which seem sometimes to conflict with each other, must be clarified, confirmed, and/or reconciled by forthcoming studies expected in the near future. To enable the intricate microbial manipulation of host reproduction by CI, sophisticated and finely tuned interactions must take place across the symbiont proteins and the host cellular components. Do CifA and CifB act on host elements separately or hand in hand? Do Cif proteins firmly stick to paternal chromosomes or merely leave a footprint for modification? How quickly and with what timing do CifA and/or CifB disappear from paternal and maternal germ cells? How does CifA behave in oocytes, with or without interactions with CifB, to perform rescue? Are there any pivotal coactors? Observation, description, and understanding of how the microbial proteins are dancing in the host cellular ballroom will reveal the secret of what makes *Wolbachia* the most successful endosymbiont across the enormous diversity of the arthropods.
